# Diagnostic Laparoscopy Revealed an Occult Low-Grade Appendiceal Mucinous Neoplasm Presenting as Pseudomyxoma Peritonei

**DOI:** 10.70352/scrj.cr.26-0614

**Published:** 2026-11-10

**Authors:** Moito Iijima, Saaya Nishikawa, Tomohiro Suzuki, Kei Shiraishi, Kanae Kashiwazaki, Aya Sasaki, Hiroshi Uchida

**Affiliations:** 1 Department of Obstetrics and Gynecology, Tokyo Dental College Ichikawa General Hospital, Ichikawa, Chiba, Japan; 2 Department of Obstetrics and Gynecology, National Hospital Organization Saitama Hospital, Wako, Saitama, Japan; 3 Department of Pathology and Laboratory Medicine, Tokyo Dental College Ichikawa General Hospital, Ichikawa, Chiba, Japan; 4 Department of Pathology and Laboratory Medicine, International University of Health and Welfare Ichikawa General Hospital, Ichikawa, Chiba, Japan; 5 Reproduction Center, International University of Health and Welfare Ichikawa General Hospital, Ichikawa, Chiba, Japan

**Keywords:** pseudomyxoma peritonei, low-grade appendiceal mucinous neoplasm, diagnostic laparoscopy, appendix, peritoneal malignancy

## Abstract

**INTRODUCTION:**

Pseudomyxoma peritonei (PMP) is a rare clinical condition that most commonly arises from low-grade appendiceal mucinous neoplasms (LAMNs). However, preoperative identification of the appendiceal primary lesion may be challenging when characteristic appendiceal abnormalities are absent on imaging studies. We report a rare case of PMP caused by a radiologically occult LAMN that was initially suspected to represent peritoneal carcinomatosis.

**CASE PRESENTATION:**

A 74-year-old woman with no abdominal symptoms was referred to our hospital after a pelvic mass in the pouch of Douglas was incidentally detected during a preoperative evaluation for orthopedic surgery. Serum cancer antigen 125 was mildly elevated (37.2 U/mL), whereas carcinoembryonic antigen and carbohydrate antigen 19-9 levels were within normal limits. CT and MRI demonstrated peritoneal lesions suggestive of peritoneal dissemination; however, no appendiceal abnormality was identified. Diagnostic laparoscopy was performed to obtain a definitive diagnosis. Intraoperatively, mucinous deposits were observed within the peritoneal cavity, and an appendiceal fistulous opening was identified, suggesting an appendiceal origin. The patient subsequently underwent an appendectomy, and histopathological examination revealed a LAMN.

**CONCLUSIONS:**

This case highlights the diagnostic limitations of preoperative imaging in patients with occult appendiceal neoplasms and demonstrates the value of diagnostic laparoscopy in establishing an accurate diagnosis and guiding appropriate management.

## INTRODUCTION

Pseudomyxoma peritonei (PMP) is characterized by the progressive accumulation of mucin within the peritoneal cavity and most commonly originates from low-grade appendiceal mucinous neoplasms (LAMNs).^1)^ Although CT and MRI play central roles in the preoperative evaluation of PMP, identification of the appendiceal primary lesion may be difficult in some patients.^2)^

Diagnostic laparoscopy is an established diagnostic modality for evaluating peritoneal malignancies, allowing direct assessment of peritoneal disease and acquisition of tissue for histopathological diagnosis when imaging findings are inconclusive.^3,4)^

Here, we report a case of a radiologically occult LAMN that was initially suspected to represent peritoneal carcinomatosis and was correctly diagnosed by diagnostic laparoscopy.

## CASE PRESENTATION

A 74-year-old woman was referred to our hospital after a pelvic mass in the pouch of Douglas was incidentally detected during a preoperative examination for orthopedic surgery at another hospital. She was asymptomatic. Laboratory investigations revealed a mildly elevated serum cancer antigen 125 (CA125) level of 37.2 U/mL, whereas carcinoembryonic antigen (CEA; 2.5 ng/mL) and carbohydrate antigen 19-9 (CA19-9; 2.0 U/mL) levels were within the normal ranges.

MRI revealed a cystic lesion in the pouch of Douglas associated with localized peritoneal thickening, raising suspicion for ovarian cancer or primary peritoneal carcinoma with peritoneal dissemination (**Fig. 1A**, **1B**). Contrast-enhanced CT demonstrated a 46-mm pelvic mass adjacent to the uterus accompanied by a moderate amount of pelvic ascites (**Fig. 1C**). No enlargement or cystic lesion of the appendix was identified on CT (**Fig. 1D**). The appendiceal primary lesion was not identified on preoperative MRI. Upper and lower gastrointestinal endoscopy revealed no evidence of a primary gastrointestinal malignancy.

**Fig. 1:**
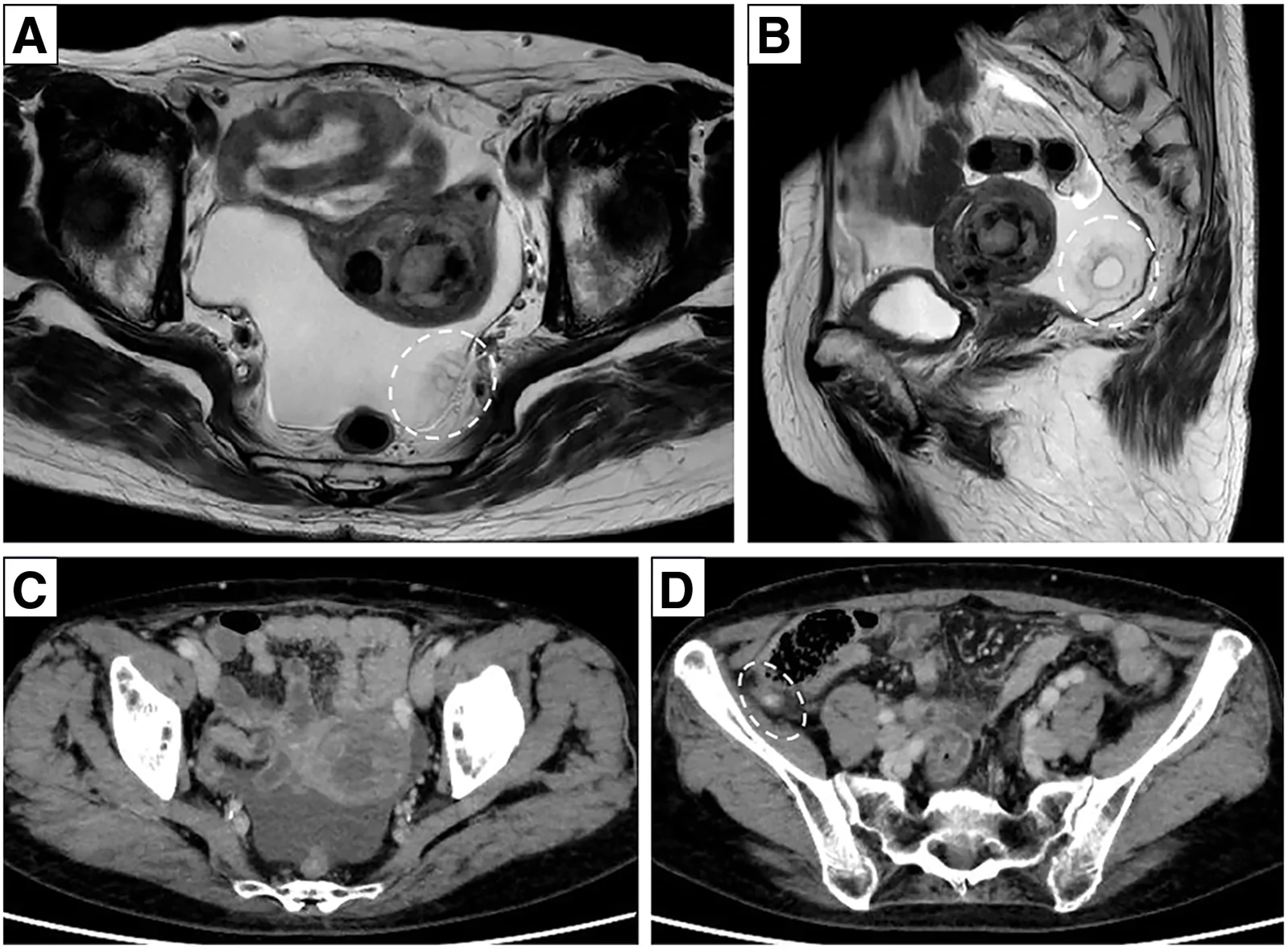
Preoperative radiological findings. (**A**, **B**) Pelvic MRI demonstrated a cystic lesion (dashed circles) in the pouch of Douglas with localized peritoneal thickening. (**C**) Contrast-enhanced CT demonstrated a pelvic mass associated with a moderate amount of ascites. (**D**) CT demonstrated no obvious appendiceal abnormality (dashed circle), including appendiceal enlargement or a cystic lesion.

Diagnostic laparoscopy was performed to establish a definitive diagnosis. Intraoperatively, gelatinous mucin was found predominantly within the pelvis, with a small amount surrounding the appendix (**Fig. 2A**). No obvious lesions were identified on the liver surface, omentum, small bowel, or small-bowel mesentery. A peritoneal biopsy obtained at the beginning of the procedure was submitted for frozen-section examination; however, no tumor was identified. Because ovarian cancer or primary peritoneal carcinoma was suspected preoperatively and a uterine leiomyoma was also present, a total hysterectomy and bilateral salpingo-oophorectomy had been planned and were subsequently performed. During the hysterectomy, a peritoneal nodule was identified in the pouch of Douglas and was excised for a second frozen-section examination (**Fig. 2B**). Histopathological examination revealed a mucinous tumor, and if the lesion was of gynecologic origin, a tumor of at least borderline malignancy was suspected. Therefore, a partial omentectomy was additionally performed. Subsequent careful inspection of the appendix revealed a grossly unremarkable appendix with a small amount of adherent gelatinous mucin (**Fig. 2C**). Further inspection of the appendiceal tip identified a fistulous opening with adherent mucin (**Fig. 2D**), raising suspicion of an appendiceal origin of the disease. An intraoperative consultation with the general surgery team was requested, and an appendectomy was subsequently performed during the same operation. Ascitic fluid was also collected for cytological examination.

**Fig. 2:**
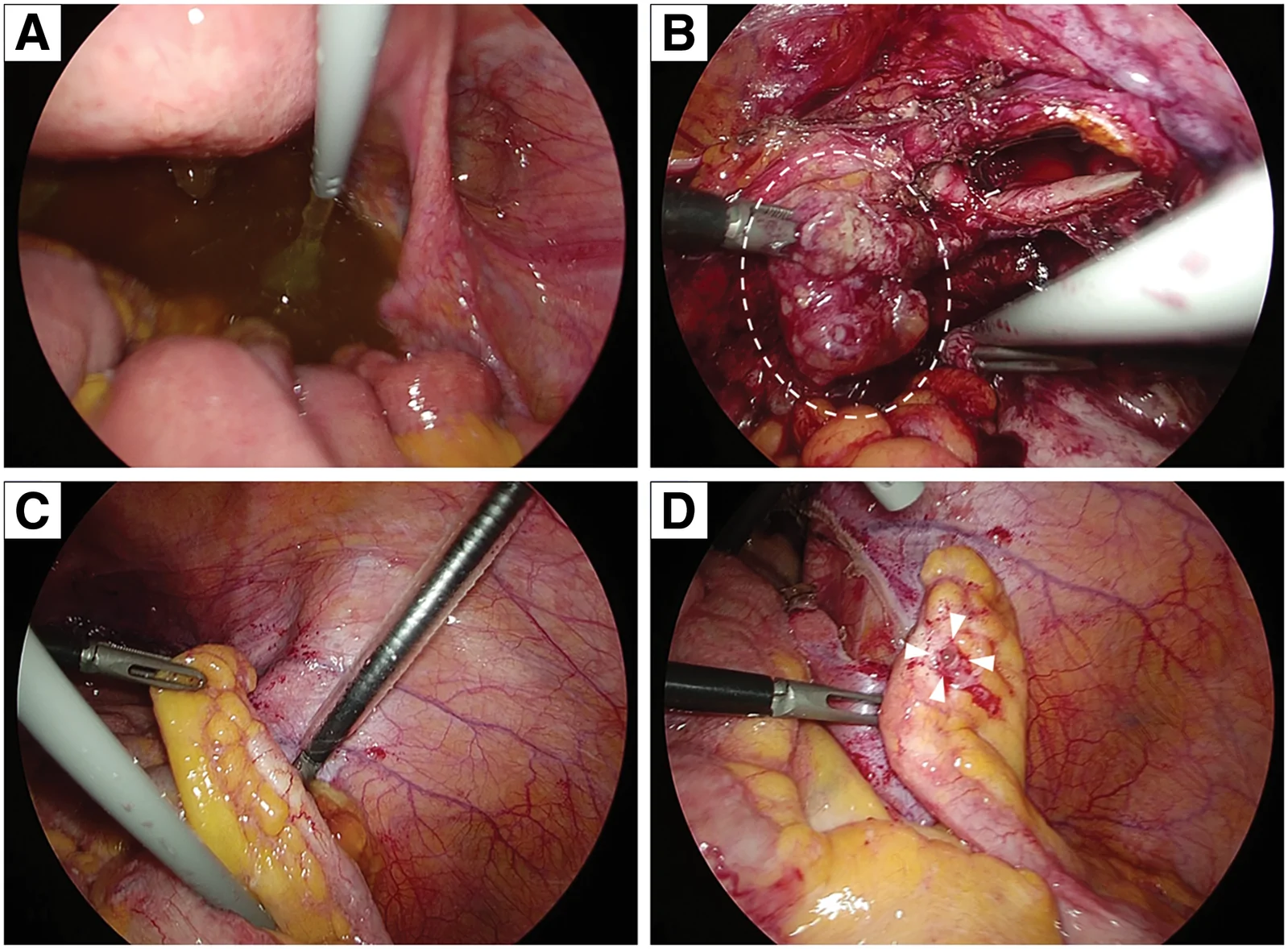
Intraoperative laparoscopic findings. (**A**) Gelatinous mucin occupying the pelvic cavity. (**B**) A peritoneal nodule in the pouch of Douglas (dashed circle), which was biopsied for histopathological examination. (**C**) Laparoscopic view of a grossly unremarkable appendix with a small amount of adherent gelatinous mucin. (**D**) A fistulous opening at the appendiceal tip (arrowheads) with adherent mucin, suggesting an appendiceal origin of the pseudomyxoma peritonei.

Histopathological examination of the appendix revealed a LAMN (**Fig. 3A**, **3B**). At the appendiceal tip, perforation of the appendiceal wall was identified, corresponding to the fistulous opening observed intraoperatively (**Fig. 3C**). The neoplasm extended into the subserosal tissue, and mucin was present on the serosal surface. Based on the presence of serosal mucin, the appendiceal lesion was classified as pT4a. Histopathological examination of the peritoneal lesion revealed mucin-containing low-grade neoplastic mucinous epithelial cells. According to the Peritoneal Surface Oncology Group International classification, the peritoneal lesion was classified as low-grade mucinous carcinoma peritonei (**Fig. 3D**). Ascitic fluid cytology demonstrated atypical glandular cell clusters in abundant extracellular mucin and was reported as suspicious for malignancy. No tumor involvement was identified in the uterus, bilateral adnexa, or omentum. The postoperative course was uneventful. The patient was followed postoperatively by both the Department of Gynecology and the Department of Surgery. Gynecologic follow-up was completed 3 months after surgery, while surveillance was continued by the Department of Surgery without additional treatment. Follow-up CT and tumor marker measurements showed no evidence of disease progression during the 8 months after surgery. The patient was subsequently referred to another hospital for continued surveillance.

**Fig. 3:**
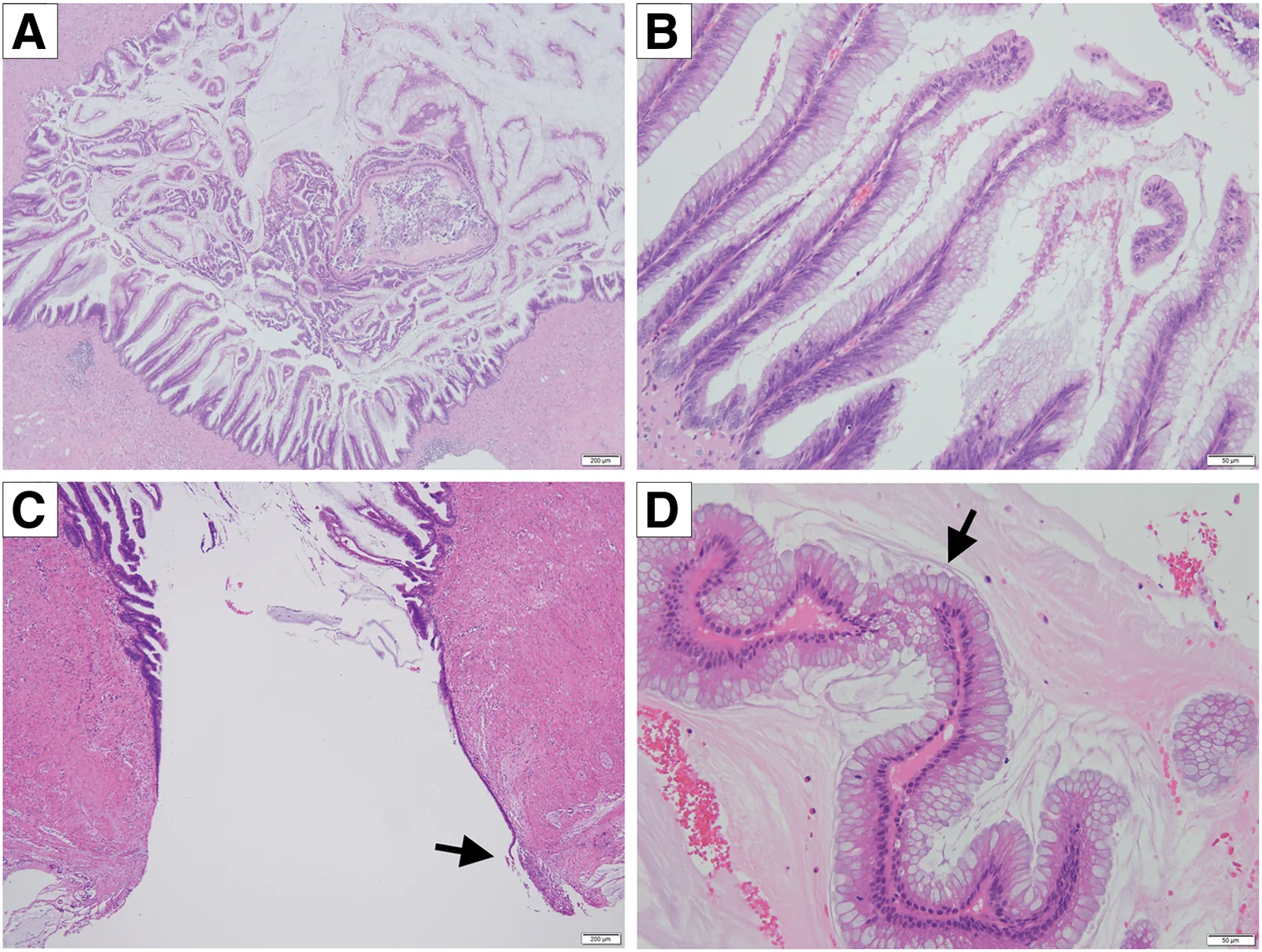
Histopathological findings of the appendix and peritoneal lesion (H&E). (**A**) Low-power view of the appendix demonstrating a low-grade appendiceal mucinous neoplasm (scale bar, 200 μm). (**B**) High-power view showing mucinous epithelium with low-grade cytologic atypia (scale bar, 50 μm). (**C**) Histopathological section of the appendiceal tip showing perforation of the appendiceal wall, corresponding to the fistulous opening identified intraoperatively. The arrow indicates extension of the neoplasm into the subserosal tissue (scale bar, 200 μm). (**D**) Histopathological examination of the peritoneal lesion showing low-grade neoplastic mucinous epithelial cells (arrow) within abundant extracellular mucin, consistent with low-grade mucinous carcinoma peritonei according to the Peritoneal Surface Oncology Group International classification (scale bar, 50 μm). H&E, hematoxylin and eosin

## DISCUSSION

PMP most commonly originates from LAMNs.^1)^ Contrast-enhanced CT and MRI are the primary imaging modalities for the preoperative evaluation of PMP. However, identification of the appendiceal primary lesion may be difficult, particularly in patients with established peritoneal disease.^2)^ The present case clearly illustrates this diagnostic limitation. Despite the presence of PMP, no appendiceal primary lesion was identified on either CT or MRI, making preoperative identification of the primary lesion difficult. Furthermore, upper and lower gastrointestinal endoscopy failed to identify the primary lesion.

Diagnostic laparoscopy plays an important role in evaluating patients with suspected peritoneal malignancy when imaging findings are inconclusive. It enables systematic inspection of the peritoneal cavity, accurate assessment of the extent of disease, and biopsy of suspicious lesions for histopathological diagnosis.^5–7)^ In the present case, despite a comprehensive preoperative evaluation, including CT, MRI, and gastrointestinal endoscopy, the primary lesion could not be identified. Careful inspection of the appendiceal tip revealed a focal abnormality suggestive of an appendiceal origin. This finding prompted an intraoperative consultation with the general surgery team, and an appendectomy was performed during the same procedure, ultimately leading to the diagnosis of LAMN-associated PMP. These findings suggest that careful inspection of the appendix should be incorporated into diagnostic laparoscopy for patients with suspected peritoneal malignancy when the primary lesion cannot be identified preoperatively.

Initially, based on the mildly elevated CA125 level and the preoperative imaging findings, ovarian cancer or primary peritoneal carcinoma was considered the most likely diagnosis. However, histopathological examination revealed that the PMP originated from a LAMN. During laparoscopy, a fistulous opening at the appendiceal tip with adherent mucin was identified, indicating an appendiceal origin of the disease. Histopathological examination confirmed perforation of the appendiceal wall at the appendiceal tip, corresponding to the fistulous opening identified intraoperatively. This case emphasizes that an appendiceal origin should be considered in patients with suspected peritoneal malignancy even when preoperative imaging fails to demonstrate an appendiceal primary lesion.

Previous reports have highlighted the importance of evaluating the appendix in patients with peritoneal disease of unknown origin. Sugarbaker reported 2 cases of occult appendiceal adenocarcinoma that were not identified by radiological or endoscopic examinations and emphasized the importance of visualizing the entire appendix during surgical exploration^7)^. Similar cases of appendiceal mucinous neoplasms mimicking gynecologic malignancies have also been reported.^8,9)^ In the present case, however, no appendiceal lesion was identified on preoperative imaging, and the appendix initially appeared grossly unremarkable during laparoscopy. Only detailed inspection of the appendiceal tip revealed a subtle fistulous opening with adherent mucin, leading to appendectomy and the diagnosis of LAMN-associated PMP. Thus, the present case further emphasizes that, in patients with PMP of uncertain origin, the entire appendix should be carefully inspected even when its initial intraoperative appearance is unremarkable.

Cytoreductive surgery (CRS) with hyperthermic intraperitoneal chemotherapy (HIPEC) is generally considered the standard treatment strategy for resectable PMP.^6)^ In the present case, postoperative surveillance without additional treatment was selected by the surgical team. However, whether CRS with HIPEC was specifically considered could not be determined retrospectively from the available medical records. The lack of documented information regarding the decision-making process for postoperative management, including consideration of CRS with HIPEC, is a limitation of this report.

## CONCLUSIONS

This case highlights the diagnostic value of laparoscopy in patients with suspected peritoneal malignancy when the primary lesion cannot be identified by preoperative imaging. Careful inspection of the appendix during laparoscopy may facilitate the diagnosis of an occult LAMN presenting as PMP.

## DECLARATIONS

### Availability of data and materials

The data supporting the findings of this article are included within the article.
